# Supplementary material for: Effect of rhythmic auditory stimulation (RAS)® with and without melody on Parkinson’s disease (PD) patients with deep brain stimulation (DBS): A study protocol
Source: PLoS One. 2026 Mar 18;21(3):e0344290. doi: 10.1371/journal.pone.0344290 (PMC12998828; doi:10.1371/journal.pone.0344290)
Supplement: S1 File — (PDF) [file pone.0344290.s002.pdf]

Happy March version

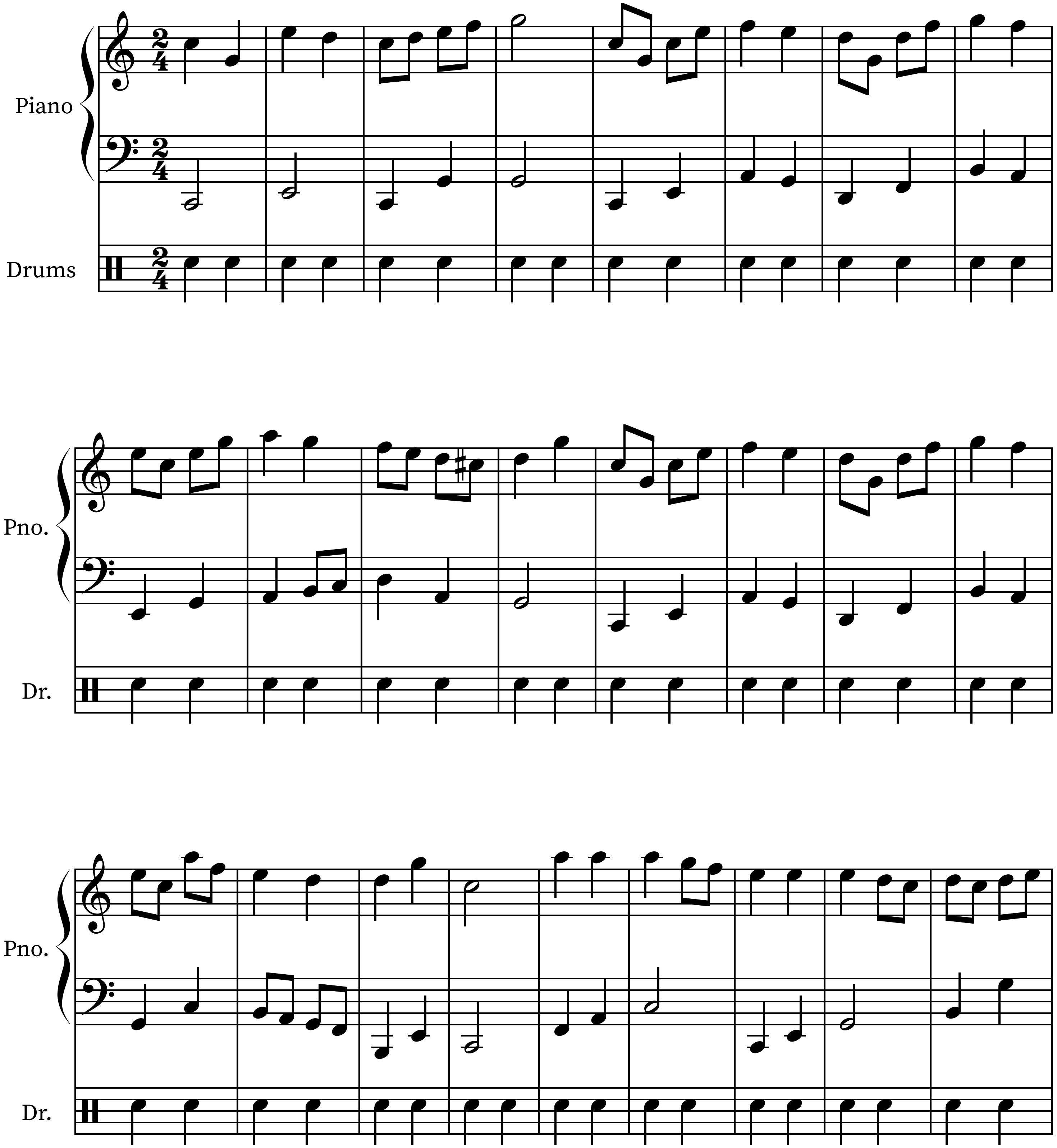

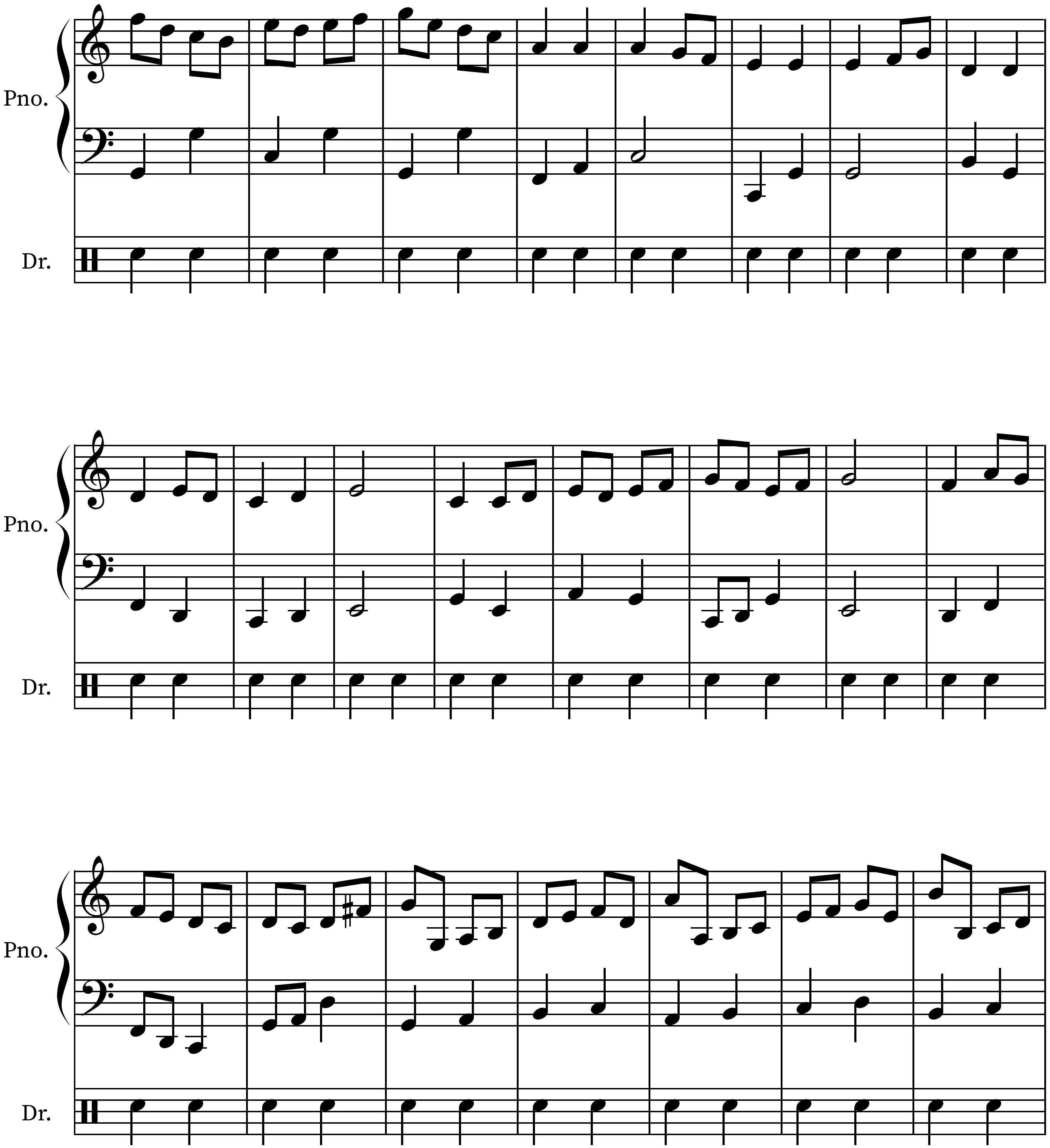
Pno.
Dr.
Pno.
Dr.
Pno.
Dr.

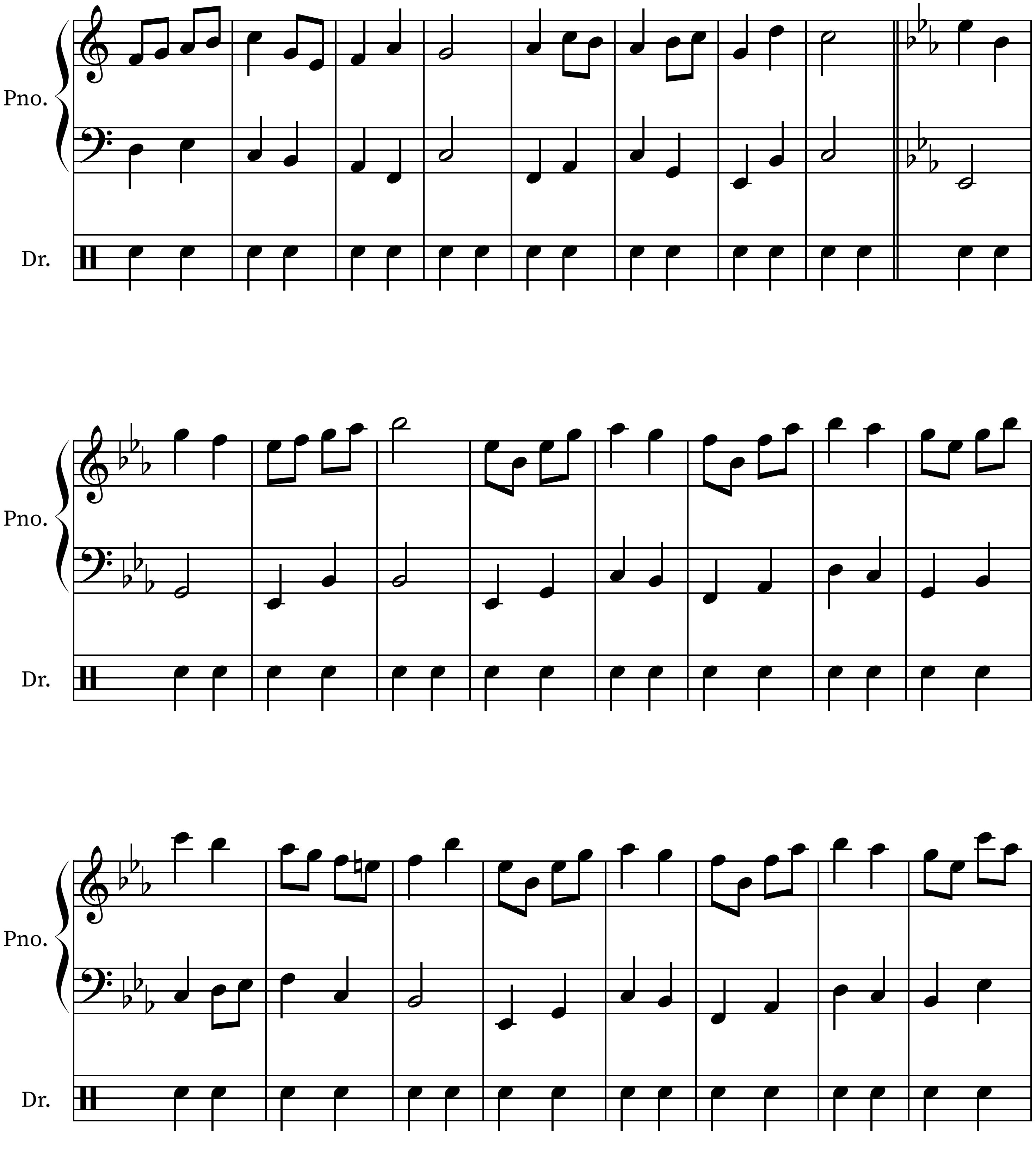
Pno.
Dr.
Pno.
Dr.
Pno.
Dr.

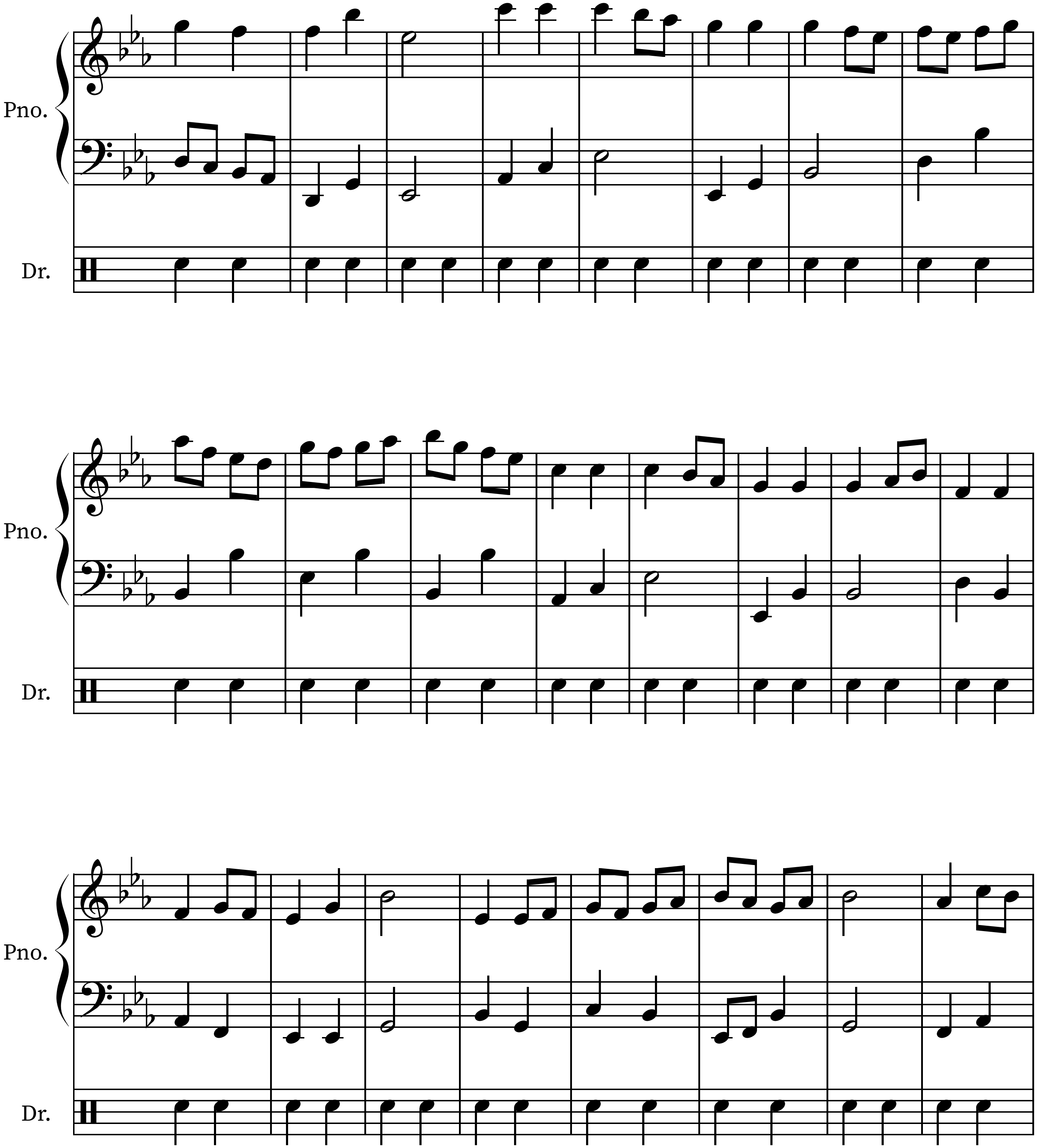
Pno.
Dr.
Pno.
Dr.
Pno.
Dr.

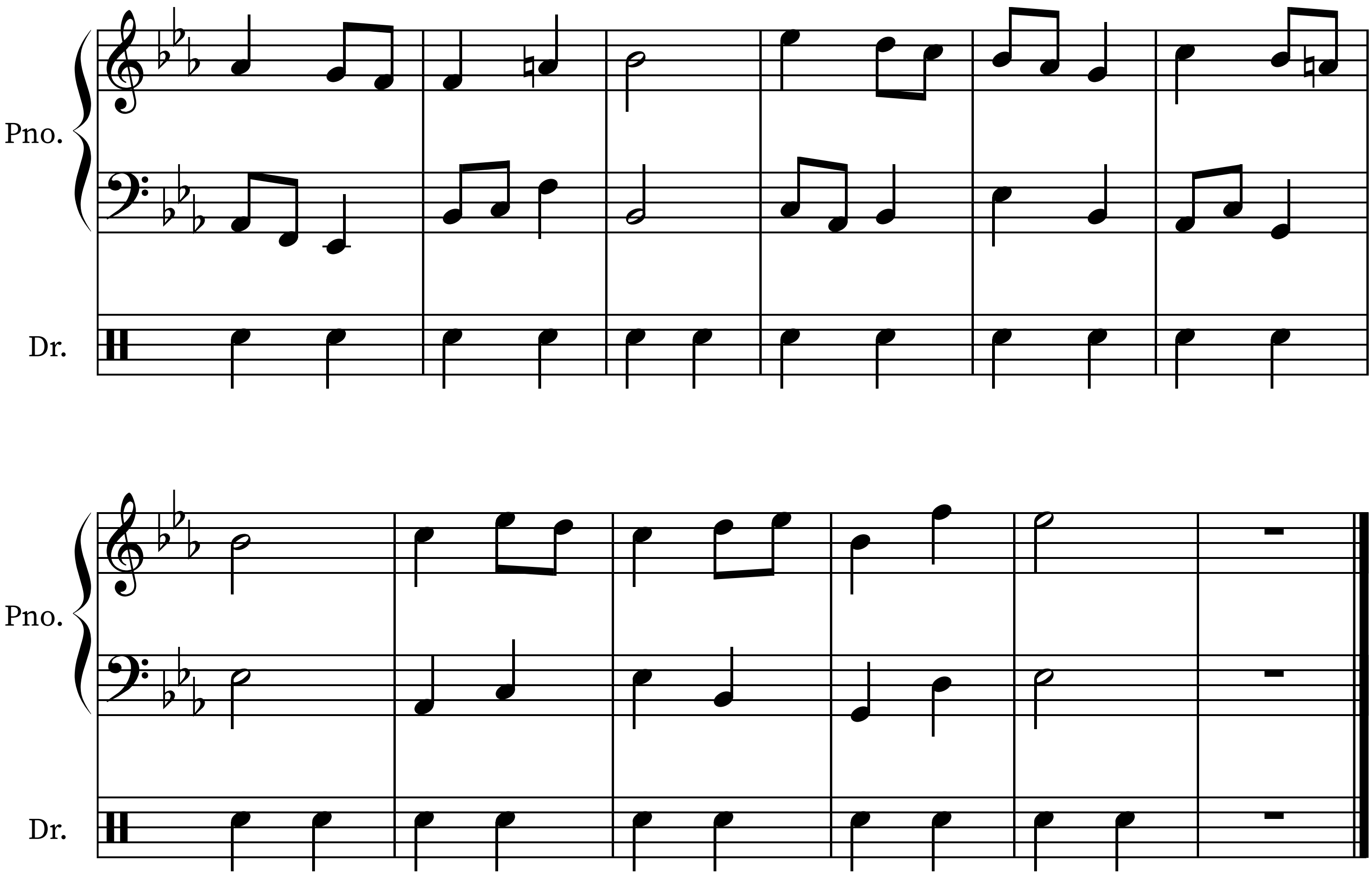
Pno.
Dr.
Pno.
Dr.
