# Supplementary material for: Effect of rhythmic auditory stimulation (RAS)® with and without melody on Parkinson’s disease (PD) patients with deep brain stimulation (DBS): A study protocol
Source: PLoS One. 2026 Mar 18;21(3):e0344290. doi: 10.1371/journal.pone.0344290 (PMC12998828; doi:10.1371/journal.pone.0344290)
Supplement: S3 File — (PDF) [file pone.0344290.s004.pdf]

JHM IRB - eForm A – Protocol

- **Use the section headings to write the JHM IRB eForm A, inserting the appropriate material in each. If a section is not applicable, leave heading in and insert N/A.**
- **When submitting JHM IRB eForm A (new or revised), enter the date submitted to the field at the top of JHM IRB eForm A.**

****

1. Abstract

Parkinson Disease (PD) is a progressive neurodegenerative disorder that impacts both motor function (including tremor, rigidity, bradykinesia, and gait difficulty/postural instability) and nonmotor function (e.g., cognitive and mood impairments) (Armstrong & Okun, 2020).

Deep brain stimulation (DBS) has been used to manage motor fluctuations or complications of dopamine replacement therapy in patients with PD (Pötter-Nerger & Volkmann, 2013; Quinn et al., 2015). The Medtronic Percept™ PC DBS device is the first commercially available platform for patients with movement disorders that is capable of in vivo brain oscillation recording. This allows for simultaneous delivery of electrical simulation and recording of the local field potentials.

Rhythmic auditory stimulus (RAS) is a Neurologic Music Therapy (NMT) technique that utilizes an auditory rhythmic cue to entrain gait to a specific rhythm. RAS, as an anticipatory time cue, can be used as both an immediate entrainment stimulus, providing rhythmic cues during movement, and as a facilitating stimulus for planning and executing movements to achieve more functional gait patterns. (M. Thaut & Hoemberg, 2014). The mechanism by which RAS exerts its effects in PD is unclear. It has been suggested that RAS can bypass the impaired frontostriatal gait networks in PD by recruiting cerebellothalamocortical networks; alternatively (or concurrently), RAS may augment the function of those frontostriatal networks (Koshimori & Thaut, 2018) .

Given the separate lines of evidence that RAS and DBS can help improve gait patterns, we propose this study to understand the behavioral and neurophysiological mechanisms of RAS and explore the clinical effects of RAS on DBS in patients with PD. To further explore the potential of music and deepen the understanding of RAS, this study will also be supplemented by a phase involving RAS with melody. The goal is to potentially develop a new tool to reinforce the effect of RAS.

The protocol will consist of two parts (DBS OFF and DBS ON). The order of stimulation states will be randomly assigned to the participants. During DBS ON, participants will receive either their previously optimized stimulation or a calibrated stimulation that mimics their clinically optimized stimulation as closely as possible if the exact stimulation is not possible due to limitations of the Medtronic Device after a 10-minute wash-in/washout period. The researchers will measure participants' gait parameters (cadence, velocity, and stride length) with a 2-minute walk (a set distance of 10 meters during the 2-minute walk) and gait patterns using relevant items from the MDS-UPDRS-III rating scale during stimulation ON (Pre-RAS). The pre-RAS phase will be followed by an RAS intervention phase. The participants will then walk to the metronome beats for eight minutes (4 minutes for the same beat as baseline cadence and 4 minutes for 10% faster than baseline cadence) (RAS), and their gait parameters will be recorded. Within each set tempo, the participants will walk for 2 minutes to a pure metronome beat followed by a 2-minute walk with melody set to the same tempo. To counterbalance the effect of having the melody walk first, the order in which the participants firswalk with

melody or without melody will also be randomly assigned. Between each of the 2-minute walks will be a 1-minute break. Finally, immediately after this walking bout, the same assessment as for Pre-RAS will be conducted (Post-RAS). Electrophysiological activity (local field potentials, LFPs) will be collected across all stages (pre, during, and post-RAS) of evaluation.

In DBS OFF, there will be a separate 10-minute washout period if it is taking place after DBS ONs so that the participant's brain circuits can adjust to not being stimulated. Except for the DBS stimulation state, DBS OFF will follow the same protocol as DBS ON above.

2. Objectives

This study seeks to investigate the mechanisms of behavioral and neurophysiological rhythmic entrainment using RAS with and without melody for gait patterns in patients with PD who already have the Percept^{TM} PC DBS device implanted (subthalamic nucleus, STN, and internal globus pallidus, GPi).

Primary goal:

A. Comparison of gait parameters (cadence, velocity, and stride length) before, during, and after RAS in DBS ON and DBS OFF state.
B. Comparison of MDS-UPDRS-III (items 3.9-13) scores in before, during, and after RAS in each DBS ON and OFF state.
C. Comparison of power spectrum density of Local Fields Potential (LFP) (micro-volts-squared per Hz) in before, during, and after RAS in each DBS ON and OFF state.
D. Comparison of goals A, B, and C with and without melody.

3. Background

a. Motor Effects of STN-DBS

DBS of the STN (STN-DBS) in Parkinson disease is superior to medical therapy in treating motor symptoms and improving quality of life despite the adverse events associated with DBS (Weaver et al., 2009). However, although STN-DBS has been effective in improving some gait measures reflecting hypokinesia, it has not been uniformly successful in improving certain temporal gait factors and has not always improved gait freezing. Furthermore, given the gradual decline over time in STN-DBS' effectiveness on gait disturbances, a multidisciplinary approach including optimized rehabilitation therapy is warranted (Pötter-Nerger & Volkmann, 2013).

b. Motor Effects of GPi-DBS

DBS of the GPi (GPi-DBS) has also been shown to improve symptoms and quality of life. While the differences between STN-DBS and GPi-DBS are still being researched, some studies have suggested that STN-DBS allows for greater medication reduction, whereas GPi-DBS may lead to a more direct suppression of dyskinesia without the need to reduce medication (Vitek 2022). Some studies also suggest that GPi leads to a greater suppression of psychiatric symptoms (Liu et al., 2014). Similar to STN-DBS, there has been no recorded uniform success in gait improvement, suggesting a need for a multidisciplinary therapy approach.

c. RAS

Rhythmic auditory stimulus (RAS) is a Neurologic Music Therapy (NMT) technique that utilizes an auditory rhythmic cue to entrain gait to a specific rhythm. RAS, as an anticipatory time cue, can be used as both a short-term immediate entrainment stimulus, providing rhythmic cues during movement, and as a facilitating stimulus for planning and executing a movement to achieve more functional gait patterns. Cadence, gait velocity, and stride length are the commonly used parameters to monitor changes in a patient's gait. (M. Thaut & Hoemberg, 2014).

RAS has been shown to effectively improve gait velocity, cadence, stride length, and potentially decrease falls in patients with PD (Hausdorff et al., 2007; Pau et al., 2016; M. H. Thaut et al., 2019).

Several mechanisms have been proposed for the therapeutic effects of rhythmic cues in patients with PD. Due to impairments of the basal ganglia and loss of frontostriatal connectivity, patients with PD experience a loss of rhythm perception and timing, resulting in various gait abnormalities in PD, including freezing of gait, decreased stride length and gait velocity, and a rhythmically unstable walking pace (Koshimori & Thaut, 2018). External auditory cueing may activate cerebello-thalamic networks that are generally spared in PD and this may compensate for the PD-associated basal ganglia abnormalities (Koshimori et al., 2019; M. Thaut & Hoemberg, 2014). In addition, it has been hypothesized that the beneficial effects of rhythmic cues may be partly due to modulation of brain activity in the pedunculopontine nucleus (PPN) (Molina et al., 2020). Another possible mechanism for rhythmic cueing in parkinsonism involves rhythmic entrainment: motor synchronization with the auditory system, which remains relatively intact in PD (Matsumoto et al., 2014). Phase-locking values in the beta and gamma frequency bands in PD did not significantly differ from those in healthy persons, indicating that auditory entrainment is still present in PD (Buard et al., 2019). There are some discrepancies in the results, though, as another study that looked at PD patients revealed auditory entrainment in the alpha and beta frequency bands (Te Woerd et al., 2017). To better understand the brain mechanisms behind the positive benefits of rhythmic auditory cuing on motor behaviors in PD, additional research into auditory entrainment is necessary.

d. UPDRS-III Rating & Testing

As a standard protocol to assess the severity of symptoms associated with PD, Movement Disorders Society - Unified Parkinson's Disease Rating Scale (MDS-UPDRS) – III is implemented either during clinic visit or during DBS electrode implantation operation. The purpose MDS-UPDRS-III is to 1) assess the baseline severity of the PD symptoms for subsequent therapeutic intervention 2) to evaluate the clinical efficacy of DBS or other treatments.

Symptoms assessed from MDS- UPDRS-III are categorized across multiple domains in cognitive, mood, behavioral, and motor. The scale ranges from 0 – 4, with a higher scale representing higher severity. Specifically, the motor symptoms of PD, such as bradykinesia, dyskinesia, gait, and tremor, are evaluated using a series of clinical tests.

4. Study Procedures

a. Study design, including the sequence and timing of study procedures (distinguish research procedures from those that are part of routine care).

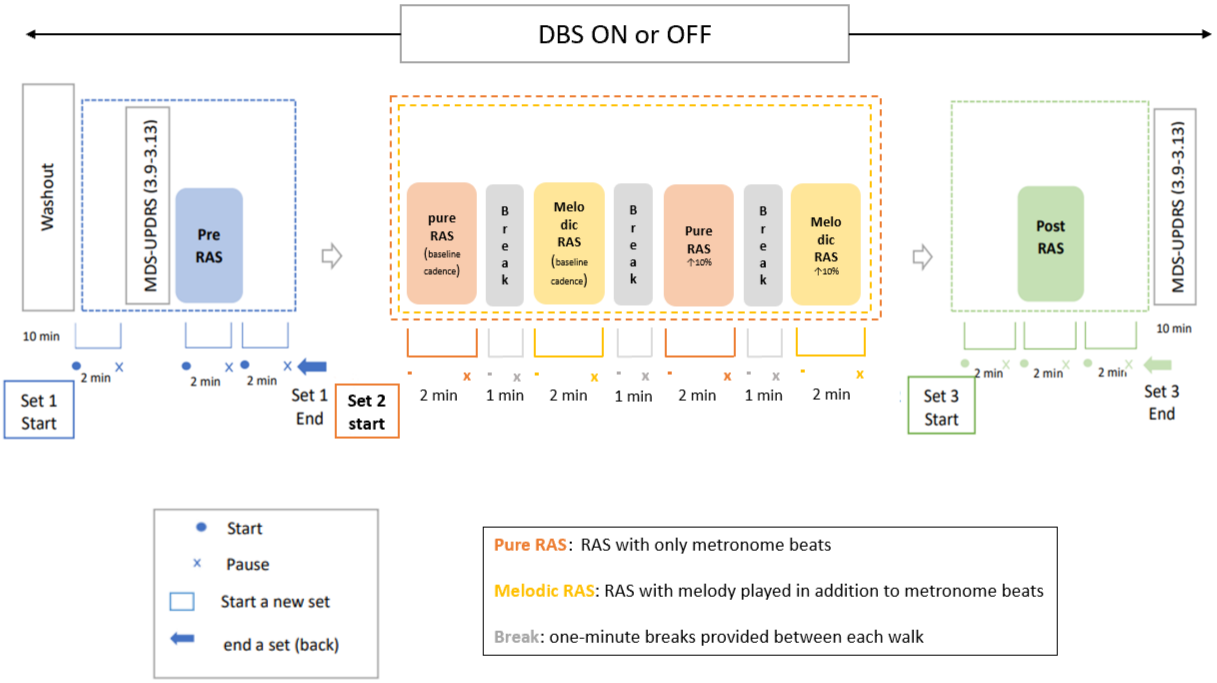

Figure 1. Study Protocol

Participants who complete the consent and enrollment process will either remain for an additional up to 90 minutes following their routine clinic visit at the Johns Hopkins Outpatient Clinic or at a later time point following their routine clinic visit

The protocol will consist of two parts (DBS ON and DBS OFF). The order of stimulation states will be randomly assigned to the participants.

Figure 1 indicates the study protocol. As part of DBS ON, participants will receive their previous clinically optimized stimulation or a stimulation that mimics their clinically optimized stimulation as closely as possible if the exact stimulation is not possible due to limitations of the Medtronic Device. during a 10-minute wash-in period. The researcher will measure participants' gait parameters (cadence, velocity, and stride length) with a 2-minute walk (a set distance of 10 meters during 2-minute walk) and gait patterns using the MDS-UPDRS-III rating scale during stimulation ON or OFF (**Pre-RAS**). The participants will then walk to the metronome beats for four minutes **RAS**) (2 minutes for the same beat as baseline cadence without melody and 2 minutes at baseline cadence 35 with melody), and their gait parameters will be recorded. They will then perform the same task at 10% faster than the baseline cadence and their gait parameters will be recorded. Between each of the 2-minute walks will be a 1-minute break. To counterbalance the effect of having the melody walk first, the order in which the participants first walk with melody or without melody will also be randomly assigned. Finally, the same assessment as the Pre-RAS will be conducted (**Post-RAS**). Electrophysiological activity (e.g., local field potentials, LFPs) will be collected across all stages (pre, during, and post-RAS).

In DBS OFF, there will be another 10-minute washout period so that the participant's brain circuits can adjust to not being stimulated. Except for the stimulation state, DBS OFF will follow the same protocol as DBS ON.

The data captured during triggered events is stored on the device and then can be downloaded in a .json file that can be imported into Matlab for analysis. The data are downloaded from the implantable pulse generator (IPG) to the clinician programmer. This .json file is then downloaded and can be stored on a secure location like OneDrive.

In the course of the assessments and the study protocol, we will record participants' gait and movement patterns. Any recordings will not be used for advertising or non-study related purposes.

We will also obtain participants music reward level using the Barcelona Music Reward Questionnaire

The patients will also be asked to fill out a post-session survey after completion of the study comparing the pure RAS vs melodic RAS interventions.

b. If your study involves data/biospecimens from participants enrolled under other research studies with a written consent or under a waiver of consent, please list the IRB application numbers for those studies.

   No biospecimens from participants in other studies will be used.

c. Study duration and number of study visits required of research participants.

   Number of visits: The study duration will either be held around 90 minutes immediately following each scheduled clinic visit or at a later time point. No additional study visits are required.

   Study duration (Figure 1):

- Before the study protocol: Barcelona Music Reward Questionnaire (2 minutes)
- Part 1: up to 4 minutes (including device setup)
  - Washout – 10 minutes
  - MDS-UPDRS III (section 3.9-13) – 2 minutes
  - 2 minute-walk – 2 minutes
  - Break – 1 minute
  - 2minute-walk with metronome beats only at base cadence
  - Break – 1 Minute
  - 2-minute walk with metronome beats and melody at base cadence
  - Break – 1 Minute
  - 2-minute walk with metronome beats only at 110% base cadence
  - Break – 1 Minute
  - 2-minute walk with metronome beats and melody at 110% base cadence
  - Break – 1 minute
  - MDS-UPDRS III (section 3.9-13) – 2 minutes
  - 2 minute-walk – 2 minutes
- Part 2: up to 40 minutes (including device setup)
  - Washout – 10 minutes

- MDS-UPDRS III (section 3.9-13) – 2 minutes
- 2 minute-walk – 2 minutes
- Break – 1 minute
- 2minute-walk with metronome beats only at base cadence
- Break – 1 Minute
- 2-minute walk with metronome beats and melody at base cadence
- Break – 1 Minute
- 2-minute walk with metronome beats only at 110% base cadence
- Break – 1 Minute
- 2-minute walk with metronome beats and melody at 110% base cadence
- Break – 1 minute
- MDS-UPDRS III (section 3.9-13) – 2 minutes
- 2 minute-walk – 2 minutes

d. Blinding, including justification for blinding or not blinding the trial, if applicable.

Participants are blinded to the stimulation state (OFF or ON). Given the significant bias introduced by the patient knowing his/her/their simulation status during the testing and generally wanting to perform "better" when their DBS system is activated, blinding/masking is required to capture the unbiased effect of RAS and/or stimulation.

e. Justification of why participants will not receive routine care or will have current therapy stopped.

The participants will have their DBS neurostimulators turned OFF and during washout period for approximately 35 minutes (washout period and testing period in DBS OFF state). Participants with DBS not infrequently experience their stimulators being turned OFF during routine clinical programming visits, and in the worst-case scenario, would NOT revert to having more prominent symptoms of Parkinson's disease that they would experience had they never undergone this elective procedure. Participants with DBS routinely turn their DBS systems off for surgical procedures, MRI's, and during clinical reprogramming sessions. The study requires this exposure in order to better understand the behavioral and neurophysiological mechanisms of RAS and DBS in improving patients' gait patterns. Participants will be closely observed by study investigators and DBS specialist throughout the visit and can be reactivated immediately if any uncomfortable symptoms reemerge.

In some cases, the participants will require a DBS ON stimulation group that differs from their clinically optimized stimulation. In order to enable the recording capabilities of the Medtronic device, the sensing leads must be set above and below the stimulating leads. If the participants' clinically optimized stimulation involves the middle leads, these exact same stimulation(s) can be used in the sensing mode. If not, the participant will receive a calibrated stimulation that mimics the clinically optimized stimulation as closely as possible. The participants will be monitored closely by both their clinician and the study clinician to ensure this difference is tolerated.

f. Justification for inclusion of a placebo or non-treatment group.

Participants will be compared using their with and without RAS in DBS on/off state (within-subject comparison), thus, a placebo or non-treatment group will not be necessary.

g. Definition of treatment failure or participant removal criteria.

Participants will be removed from the study immediately if they experience any untoward symptoms during or after DBS being turned off (in this case DBS will be turned back on at the previously optimized settings).

h. Description of what happens to participants receiving therapy when study ends or if a participant's participation in the study ends prematurely.

All participants will be instructed to continue their usual medications/stimulation once the study ends.

5. Inclusion/Exclusion Criteria

Inclusion criteria:
All participants will be adults (males or females) age 18 to 95 years old. In addition:

- diagnosed with idiopathic Parkinson's disease and have that diagnosis confirmed in the Johns Hopkins Parkinson's Disease and Movement Disorders Center
- underwent DBS Surgery (implanted Medtronic Percept™ PC DBS device in STN or GPi)
- fluency in English.

Exclusion criteria:

- Participants who have dementia, language impairment, or severe motor impairment to the point that they cannot comprehend or understand instructions prior to recording.
- Participants who have known, significant discomfort when DBS is turned "off", even briefly.
- Participants who, in the view of the investigators, would have severely increased parkinsonian symptoms in the DBS OFF state potentially causing intolerable discomfort or disability

6. Drugs/ Substances/ Devices

a. The rationale for choosing the drug and dose or for choosing the device to be used.

No drug or device is being studied. While the study includes patients under normal treatment with medications and brain stimulation. The participating patients already have DBS electrodes implanted for treatment of their movement disorder. We will only turn DBS off / on during the testing session, which is often done as part of clinical care during DBS reprogramming visits.

In particular, we will recruit participants implanted with the Medtronic Percept™ PC DBS device. This device is the first commercially available platform for patients with movement disorders that is capable of in vivo brain sensing. This allows us to deliver electrical simulation and record the local field potential simultaneously.

In order to enable the recording capabilities of the Medtronic device, the sensing leads must be set above and below the stimulating leads. This requires a creation of a BrainSense Group, typically during the clinic visit prior to testing. For the DBS on condition, whenever compatible, the frequency, intensity, and electrode contact of stimulation will not be changed. If the exact same stimulation is not possible due to limitations of the Medtronic device mentioned above, the participant will receive a calibrated stimulation that mimics the clinically optimized stimulation as closely as possible. The

participants will be monitored closely by both their clinician and the study clinician to ensure this difference is tolerated.

.

b. Justification and safety information if FDA approved drugs will be administered for non-FDA approved indications or if doses or routes of administration or participant populations are changed.
N/A

c. Justification and safety information if non-FDA approved drugs without an IND will be administered.
N/A

7. Study Statistics

a. Primary outcome variable.
   - Change in gait parameters (cadence, velocity, and stride length) before, during, and after RAS in each DBS ON and OFF state.
   - Change in MDS-UPDRS-III (section 3.9-13) scores before, during, and after RAS in each DBS ON and OFF state.
   - Change in power spectrum density of Local Fields Potential (LFP) (micro-volts-squared per Hz) before, during, and after RAS in each DBS ON and OFF state.
   - Comparison of goals A, B, and C with and without melody.

b. Secondary outcome variables.
N/A

c. Statistical plan including sample size justification and interim data analysis.

- <u>Sample size:</u>
We will attempt to recruit all the patients in the movement disorders clinic who meet the inclusion criteria above. Per our clinic database, we have about 10 potential participants at this time. This pilot data will provide an effect size for a larger study.

- <u>Data analysis:</u>
All outcome measurements are continuous variable (time or scores).
   - Independent variables: Stimulation state (ON, OFF), time point (pre-RAS, pure RAS, melodic RAS and post-RAS)
   - Dependent variable 1: MDS-UPDRS-III (section 3.9: Arising from chair)
   - Dependent variable 2: MDS-UPDRS-III (section 3.10: Gait)
   - Dependent variable 3: MDS-UPDRS-III (section 3.11: Freezing of gait)
   - Dependent variable 4: MDS-UPDRS-III (section 3.12: Postural stability)
   - Dependent variable 5: MDS-UPDRS-III (section 3.13: Posture).
   - Dependent variable 6: Cadence (steps/minute)
   - Dependent variable 7: Velocity (meter/minute)
   - Dependent variable 8: Stride length (meter)

- Dependent variable 9: Power spectrum density of Local Fields Potential (LFP) (micro-volts-squared per Hz)

The Shapiro-wilks test will be used to check the normality of the data.
We will use non-parametric paired-sample tests (e.g., Wilcoxon paired-sample test) as appropriate to compare the mean differences between pre-RAS, pure RAS, melodic RAS and post-RAS in each stimulation state (e.g., DBS ON-pre-RAS vs DBS ON-pure RAS vs DBS ON-melodic RAS, DBS ON-pre-RAS vs DBS ON-post-RAS, and DBS ON-pure RAS vs DBS ON-melodic RAS vs DBS ON-post-RAS; same comparison for DBS OFF state).
Further, the mean differences between DBS ON and OFF over each time period will be compared (e.g., DBS ON-pre-RAS vs DBS OFF-pre-RAS; DBS ON-pure RAS vs DBS OFF- pure RAS; DBS ON-melodic RAS vs DBS OFF- melodic RAS, DBS ON-post-RAS vs DBS OFF-post-RAS).
A False Discovery Rate (FDR) correction will be used to account for multiple comparisons (Benjamini & Hochberg, 1995).

d. Early stopping rules.

Participants will be allowed to discontinue the testing at any time, and will be informed of this right during the consent. If a patient chooses to stop at any time during the testing, data already collected will be included in this research unless he/she specified that data should be withdrawn. Given the observational nature of the study, there is no interim analysis and are no early stopping rules.

8. Risks

a. Medical risks, listing all procedures, their major and minor risks and expected frequency.

1. Loss of benefit of DBS when settings are changed during the experiment. This may allow Parkinson's (or dystonia) related stiffness, slowness of movement, or tremor to emerge. Stiffness can sometimes manifest as muscular discomfort, but this varies by patient. Essentially, the patients may return to their pre-DBS state just during the experimental period if all benefit of DBS is lost with the change in stimulation parameters. This is similar to when DBS is turned OFF for impedance checks or reprogramming during routine clinical visits.

2. Risk of falls: There will be a minimal risk of falls.

3. Clinical data breach. Clinical data about the participants are essential for answering the research question at hand. Potentially sensitive information, such as clinical diagnoses, medication dosages, and co-morbid conditions will be collected. If a data breach were to occur, this information could be available to others in the public, but the data would already have been de-identified (as described below).

4. Discomfort from MDS-UPDRS-III motor testing. Patients can sometimes experience physical fatigue while undergoing continuous motor batteries. Some patients may experience their performance on certain tasks as deficits and may feel some psychological discomfort from this.

b. Steps taken to minimize the risks.

1. To minimize the risk of discomfort from a loss of DBS efficacy when turned off, we have set the duration of the testing paradigm for “DBS-OFF” condition as at most 35 minutes so that the subject experiences the loss of benefit of DBS for limited amount of time. If severe Parkinson’s disease symptoms emerge when DBS is turned off (i.e. he/she returns to the “pre-DBS state”) and are not tolerated, we will return the participants DBS settings to his/her clinically effective programming. Symptoms should then resolve on the order of seconds to 5 minutes.

2. To minimize the risk of falls, a research team member will walk alongside the patients during the assessments and study protocol.

3. To minimize the likelihood of release of patient health information, all clinical data will be collected into the CRF’s, which will be marked with the subject’s study ID number. These CRF’s will be stored in a locked office in Carnegie 518D and only the investigator has access to this office. A copy of the list associated patient identifiers (name and DOB) with the study ID’s will be kept in a separate, locked drawer in the locked office.

4. The data from computer testing will be de-identified and only the subject’s study ID and will be used to name data files on the testing laptop, which is also locked in an office in the Carnegie building. Data files contain numerical results of behavioral performance measures and no identifying information. Any other digital data will be housed and analyzed on JH OneDrive in a folder shared only with the IRB-approved study team. Access to these storage locations can only be obtained if granted by the principal investigator and that encryption meets and exceeds standards considered to be HIPAA compliant. Data are not being shared outside of Johns Hopkins.

5. Discomfort from motor testing will be minimized by frequently assessing the participant’s comfort level and monitoring his/her mood and functional status. There are also temporary breaks built-in to the testing paradigm, with approximately 10 seconds rest per trial, and 15 second rest between each MDS-UPDRS-III test. These breaks will minimize fatigue and discomfort. If patients experience psychological discomfort from experiencing any deficits in performance, study staff will reassure them that performance is not relative to others. However, if there is still concern, the investigators will pursue treatment of cognitive or behavioral dysfunction in clinic as part of routine clinical care. The patients will be made aware during the consenting process that they can withdraw from the protocol at any time.

6. Data Safety Monitoring Plan: Monitoring will be done by the study team during the study procedure. Given that there this is not a treatment trial, there are no long-term, cumulative safety outcomes to monitor. Study team will monitor the clinical state of participants enrolled in the study, especially having DBS settings changed. Any significant discomfort that would occur from either of these conditions would be immediately reversible by changing DBS back to the clinically optimized settings. Of note, any side effects from stimulation would be similar to what the patient might experience during a typical clinical encounter for DBS programming to target PD symptoms. Changes to DBS parameters take effect within seconds to 5 minutes.

c. Plan for reporting unanticipated problems or study deviations.

Any adverse events, unanticipated problems, and/or study deviations will be reported to the IRB promptly, but no more than 10 days after discovery.

d. Legal risks such as the risks that would be associated with breach of confidentiality.

A breach of confidentiality is theoretically possible, but because this is a single-site study without transmission of data between campuses or sites, and because all data will be maintained in one secure physical and/or electronic location, breach of confidentiality is exceedingly unlikely.

e. Financial risks to the participants.

None.

9. Benefits

a. Description of the probable benefits for the participant and for society.

There is no direct benefit to the participants of the study. However, the findings of this study may contribute to a better understanding of how DBS and RAS can be applied to improve the gait patterns of individuals with Parkinson disease.

10. Payment and Remuneration

a. Detail compensation for participants including possible total compensation, proposed bonus, and any proposed reductions or penalties for not completing the protocol.

Participants will be offered a $50 honorarium for their participation in the study.

11. Costs

a. Detail costs of study procedure(s) or drug (s) or substance(s) to participants and identify who will pay for them.

There are no costs of the testing performed during this study.

References

Armstrong, M. J., & Okun, M. S. (2020). Diagnosis and treatment of Parkinson disease: A review. *Jama*, *323*(6), 548–560.

Benjamini, Y., & Hochberg, Y. (1995). Controlling the false discovery rate: A practical and powerful approach to multiple testing. *Journal of the Royal Statistical Society: Series B (Methodological)*, *57*(1), 289–300.

Buard, I., Dewispelaere, W. B., Thaut, M., & Kluger, B. M. (2019). Preliminary Neurophysiological Evidence of Altered Cortical Activity and Connectivity With Neurologic Music Therapy in Parkinson's Disease. *Frontiers in Neuroscience*, *13*, 105. https://doi.org/10.3389/fnins.2019.00105

Hausdorff, J. M., Lowenthal, J., Herman, T., Gruendlinger, L., Peretz, C., & Giladi, N. (2007). Rhythmic auditory stimulation modulates gait variability in Parkinson's disease. *European Journal of Neuroscience*, *26*(8), 2369–2375.

Koshimori, Y., Strafella, A. P., Valli, M., Sharma, V., Cho, S., Houle, S., & Thaut, M. H. (2019). Motor Synchronization to Rhythmic Auditory Stimulation (RAS) Attenuates Dopaminergic Responses in Ventral Striatum in Young Healthy Adults: [11C]-(+)-PHNO PET Study. *Frontiers in Neuroscience*, *13*, 106. https://doi.org/10.3389/fnins.2019.00106

Koshimori, Y., & Thaut, M. H. (2018). Future perspectives on neural mechanisms underlying rhythm and music based neurorehabilitation in Parkinson's disease. *Ageing Research Reviews*, *47*, 133–139. https://doi.org/10.1016/j.arr.2018.07.001

Liu Y, Li W, Tan C, Liu X, Wang X, Gui Y, Qin L, Deng F, Hu C, Chen L. Meta-analysis comparing deep brain stimulation of the globus pallidus and subthalamic nucleus to treat advanced Parkinson disease. J Neurosurg. 2014 Sep;121(3):709-18. doi: 10.3171/2014.4.JNS131711. Epub 2014 Jun 6.

Matsumoto, L., Magalhaes, G., Antunes, G. L., & Torriani-Pasin, C. (2014). Effect of rhythmic auditory cue on gait in patients with Parkinson's disease. *Revista Neurociencias*, *22*(3), 404–409.

Molina, R., Hass, C. J., Sowalsky, K., Schmitt, A. C., Opri, E., Roper, J. A., Martinez-Ramirez, D., Hess, C. W., Foote, K. D., & Okun, M. S. (2020). Neurophysiological correlates of gait in the human basal ganglia and the PPN region in Parkinson's disease. *Frontiers in Human Neuroscience*, *14*, 194.

Pau, M., Corona, F., Pili, R., Casula, C., Sors, F., Agostini, T., Cossu, G., Guicciardi, M., & Murgia, M. (2016). Effects of physical rehabilitation integrated with rhythmic auditory stimulation on spatio-temporal and kinematic parameters of gait in Parkinson's disease. *Frontiers in Neurology*, *7*, 126.

Pötter-Nerger, M., & Volkmann, J. (2013). Deep brain stimulation for gait and postural symptoms in Parkinson's disease. *Movement Disorders*, *28*(11), 1609–1615. https://doi.org/10.1002/mds.25677

Quinn, E. J., Blumenfeld, Z., Velisar, A., Koop, M. M., Shreve, L. A., Trager, M. H., Hill, B. C., Kilbane, C., Henderson, J. M., & Brontë-Stewart, H. (2015). Beta oscillations in freely moving Parkinson's subjects are attenuated during deep brain stimulation. *Movement Disorders*, *30*(13), 1750–1758.

Štillová, Klára et al. 2021. "Mozart Effect in Epilepsy: Why Is Mozart Better than Haydn? Acoustic Qualities-Based Analysis of Stereoelectroencephalography." *European Journal of Neurology* 28(5): 1463–69

Te Woerd, E. S., Oostenveld, R., de Lange, F. P., & Praamstra, P. (2017). Impaired auditory-to-motor entrainment in Parkinson's disease. *Journal of Neurophysiology*, *117*(5), 1853–1864.

Thaut, M. H., Rice, R. R., Braun Janzen, T., Hurt-Thaut, C. P., & McIntosh, G. C. (2019). Rhythmic auditory stimulation for reduction of falls in Parkinson's disease: A randomized controlled study. *Clinical Rehabilitation*, *33*(1), 34–43.

Thaut, M., & Hoemberg, V. (2014). *Handbook of neurologic music therapy*. Oxford University Press (UK).

Vitek JL. Deep brain stimulation for Parkinson's disease. A critical re-evaluation of STN versus GPi DBS. Stereotact Funct Neurosurg. 2002;78(3-4):119-31. doi: 10.1159/000068959.

Weaver, F. M., Follett, K., Stern, M., Hur, K., Harris, C., Marks, W. J., Rothlind, J., Sagher, O., Reda, D., & Moy, C. S. (2009). Bilateral deep brain stimulation vs best medical therapy for patients with advanced Parkinson disease: A randomized controlled trial. *Jama*, *301*(1), 63–73.
